# A biosensor capable of identifying low quantities of breast cancer cells by electrical impedance spectroscopy

**DOI:** 10.1038/s41598-019-42776-9

**Published:** 2019-04-23

**Authors:** L. F. E. Huerta-Nuñez, G. Gutierrez-Iglesias, A. Martinez-Cuazitl, M. M. Mata-Miranda, V. D. Alvarez-Jiménez, V. Sánchez-Monroy, Alexander Golberg, C. A. González-Díaz

**Affiliations:** 1 Escuela Militar de Graduados de Sanidad-Universidad del Ejército y Fuerza Aérea, México City, Mexico; 2 Escuela Superior de Medicina-Instituto Politécnico Nacional, México City, Mexico; 3 Escuela Nacional de Medicina y Homeopatía-Instituto Politécnico Nacional, México City, Mexico; 4 Escuela Militar de Medicina-Universidad del Ejército y Fuerza Aérea, México City, Mexico; 5 Centro Médico Naval- Secretaría de Marina, México City, Mexico; 6 Porter School of Environmental and Earth Sciences, Tel Aviv University, Tel Aviv-Yafo, Israel

**Keywords:** Diagnostic markers, Nanotechnology in cancer

## Abstract

Breast cancer (BC) is a malignant disease with a high prevalence worldwide. The main cause of death is not the primary tumor, but instead the spread of tumor cells to distant sites. The aim of the present study was to examine a new method for the detection of cancer cells in aqueous medium using bioimpedance spectroscopy assisted with magnetic nanoparticles (MNP’s) exposure to a constant magnetic field. The spectroscopic patterns were identified for three breast cancer cell lines. Each BC cell line represents a different pathologic stage: the early stage (MCF-7), invasive phase (MDA-MB-231) and metastasis (SK-BR-3). For this purpose, bioimpedance measurements were carried out at a certain frequency range with the aid of nanoprobes, consisting of magnetic nanoparticles (MNPs) coupled to a monoclonal antibody. The antibody was specific for the predominant cell surface protein for each cell line, which was identified by using RT-qPCR and flow cytometry. Accordingly, EpCAM corresponds to MCF-7, MUC-1 to MDA-MB-231, and HER-2 to SK-BR-3. Despite their low concentrations, BC cells could be detected by impedance spectroscopy. Hence, this methodology should permit the monitoring of circulating tumor cells (CTC) and therefore help to prevent recurrences and metastatic processes during BC treatment.

## Introduction

Breast cancer (BC) represents one of the biggest public health problems in the world, being the most common cause of cancer-related deaths in 103 countries (with cervix and lung cancer assuming the leading role in 43 and 27 countries, respectively)^1,2^. The great mortality rate of BC is due to the frequent lack of early diagnosis, an often poor response to chemotherapy, and the occurrence of metastasis. Less than 25% of BC patients survive more than 5 years after metastasis.

Since several studies on the process of metastasis have found cancer cells in the blood, this should certainly be an important indicator of disease dissemination as well prognosis and response to treatment^3–6^. With the ability to detect cancer cells in the blood, the progress of a given therapeutic option could be monitored and its dosage assessed in order to prevent patients from being exposed to an ineffective therapy and/or toxic doses. It has been reported that the number of cancer cells in the blood of patients diagnosed with cancer is related to prognosis and survival^7,8^. Hence, some researchers denominate the screening procedure for examining this parameter as “liquid biopsy in real time”^9^.

The distinct physical and biological properties of cancer cells provide the basis for their identification by different techniques, including flow cytometry, fluorescence activated cell sorting (FACS), gene expression studies, and so on. However, these techniques are limited by the long preparation times needed, the requirement for highly trained personnel and expensive equipment, and the lack of methodological standardization. Therefore, health care centers with limited resources need more economical methods for detecting the presence of cancer cells and evaluating the response to treatment^10^.

Karabacak *et al*. reported a complete marker-free system designed on the basis of deterministic lateral displacement, inertial focusing and magnetophoresis to sort red/white blood cells and rare cells from a whole blood sample. They pointed out that the limitation of the system is associated with immunoaffinity-based selection, critical hydrodynamic diameters, and expensive reagents and fabrication requirements^11^.

One possible approach for the detection of cancer cells in the blood is the use of nanoparticle markers that can be read by a simple spectroscopic technique. Magnetic nanoparticles (MNPs) have been widely used for *in vivo* examinations with magnetic resonance imaging, contrast enhancement, specific tissue release of therapeutic agents, hyperthermia, and magnetic field assisted radionuclide therapy^12–14^. They have also been coupled to biological materials, such as proteins, peptides, enzymes, antibodies and nucleic acid. Because of their unique properties, coupled nanoparticles can magnetically label target molecules or organelles for tracking^15^. Among the widely reviewed bioapplications of MNPs are targeted drug delivery, magnetic resonance imaging (MRI), magnetic hyperthermia/thermoablation, detection and bioseparation of bacteria, and biosensing (based on the functional materials and groups, the signals detected and the targeted receptors)^16,17^. Particularly relevant for the present study is the fact that MNPs have been coupled with antibodies to isolate cancer cells.

There are two main techniques for confirming the adequate functionalization of nanoparticles with specific molecules. Whereas the size and structure of the particles is characterized by transmission electron microscopy (TEM), the binding of MNPs to biological material is evaluated with Fourier transform infrared spectroscopy (FTIR). The latter imaging technique provides spatial information based on chemically specific IR spectra. By processing the spectral data with a variety of computational algorithms, it is possible to obtain an information-rich image of the corresponding tissue or cell type is obtained. Since the images are constructed from fingerprint spectra, they should objectively portray the underlying status of the analyzed sample^18^.

Electrical impedance spectroscopy (EIS) refers to the opposition offered by biological samples to the flow of electrical current in the frequency spectrum, which can reflect the physiological state of cells. The equivalent impedance of a single cell is comprised of the capacitance of the cell membrane and the resistance of the cytoplasm. The composition of the membrane and intracellular space also influence the electrical properties of the cell. Therefore, it possible to distinguish between tumor cells and normal cells, and even between normal cells of diverse types. Distinct types of cells show variants of electrical resistance and reactance when excited at different frequencies^19^. The many advantages of EIS in medicine and biology include its non-invasiveness, low cost, portability and ease of use. The resulting measurement from impedance spectroscopy could serve as a label-free marker for the classification of cell type^10,19–21^.

Arum Han *et al*. (2007) attempted to identify cancer cells with single cell impedance spectroscopy measurements, comparing the data from three BC cell lines and normal human breast tissue. These authors employed a microelectrical impedance spectroscopy system that traps a single cell into an analysis cavity and measures its electrical impedance over a frequency range of 100 Hz to 3.0 MHz, thus determining the impedance signature of whole cells. Clear differences were revealed in the electrical impedance between cell lines, both in magnitude and phase^22^.

Jhin-Lin Hong *et al*. (2012) introduced a microfluidic device for the measurement of single-cell impedance. The device utilizes dielectrophoretic force and electrothermal alternating current to capture a single cell and measure its impedance. This group examined four types of cell lines: two of human breast cancer (MCF-7 and MDA-MB-231), one of human cervical cancer (HeLa) and one of human lung adenocarcinoma cancer (A549). The significant differences observed in the magnitude of impedance between the cell types at a voltage ranging from 0.2 V to 1 V allowed the researchers to distinguish between the types of cancer cells tested^23^.

Impedance measurement has proven to be an effective technique for characterizing cells based on their electrical response over a particular frequency range. However, the *in vitro* detection of tumor cells in the blood still represents a considerable challenge, due to the extremely small quantity of such cells (~10–50 cells/ml)^24^. The aim of the present study was to carry out bioimpedance spectroscopy measurements to detect cancer cells in aqueous solution and identify the spectral pattern of each of three BC cell lines. The resulting fingerprint patterns should certainly be useful as a biosensor in future studies in order to identify these cells in patients. A nanoprobe (MNPs coupled to monoclonal antibodies) was used to isolate and detect the cells. The conceptual framework is based on immunomagnetic cancer cell separation from whole blood and anchoring techniques.

## Results

### EpCAM, MUC-1 and HER-2 proteins as potential targets for coupling by magnetic nanoparticles

**Figure 1:**
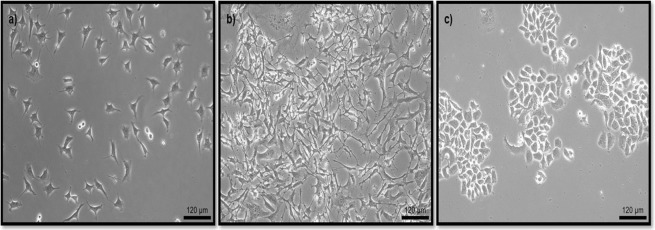
Breast cancer cell lines. (**a**) MCF-7, (**b**) MDA-MB-231 and (**c**) SK-BR-3 (Magnification 10x).

**Figure 2:**
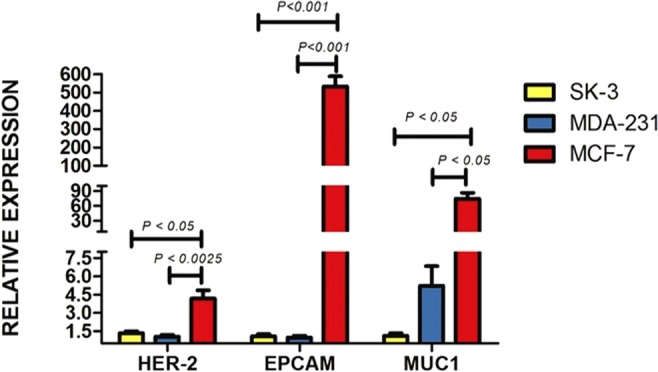
Gene expression profiling of breast cancer cell lines. Quantitative real-time PCR was employed to confirm the expression profile of *EpCAM, MUC-1* and *HER-2* in the breast cancer cell lines. Expression of *β-Actin* was used as the internal control. Data are expressed as the mean ± standard error of the mean (SEM) of three independent experiments.

**Figure 3:**
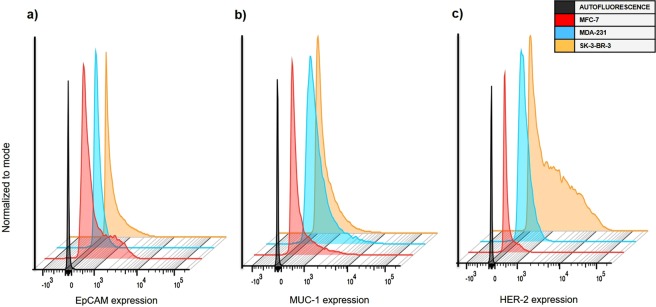
Determination cell surface protein expression. With flow cytometry, fluorescence intensity was measured in three cell lines (MCF-7, MDA-MB-231 and SK-BR-3) to evaluate the expression of the corresponding surface proteins: (**a**) EpCAM, (**b**) MUC-1 and (**c**) HER-2.

The RNA expression profile was determined for each BC cell line by RT-qPCR (Fig. 1). The highest expression of all the genes herein evaluated was found in MCF-7 cells. The gene with the greatest expression in this cell line was EpCAM (Epithelial cell adhesion molecule), whereas that in MDA-MB-231 was MUC-1 (Mucin-1). A slight non-significant difference was observed for HER-2 (Human epidermal growth factor receptor 2) in SK-BR-3 (Fig. 2). These results were confirmed by flow cytometry, which revealed a predominant protein expression of EpCAM in MCF-7, MUC-1 in MDA-MB-231 and HER-2 in SK-BR-3 (Fig. 3).

### Evaluation of the coupling of magnetic nanoparticles to antibodies by using ATR-FTIR and FTIRI

**Figure 4:**
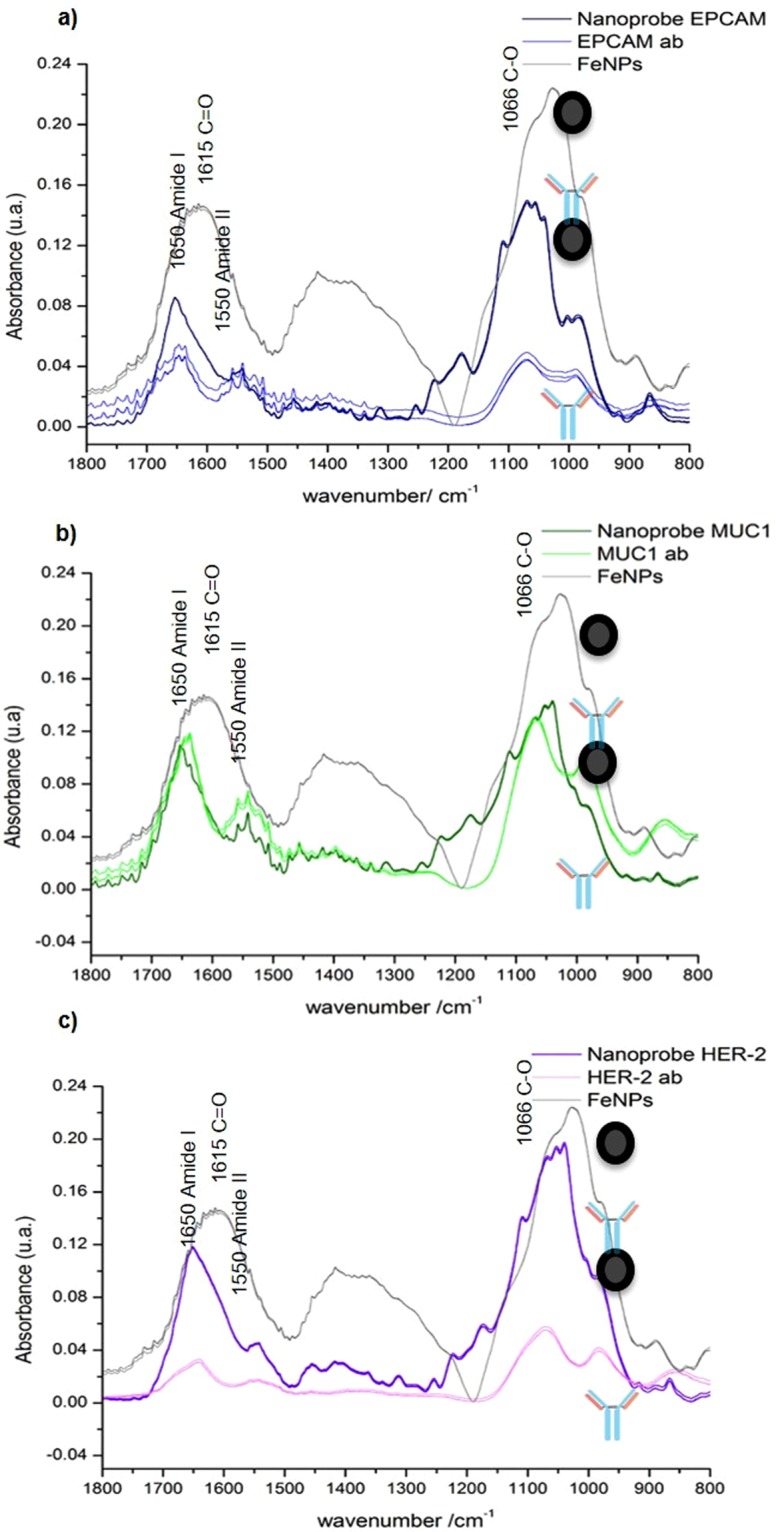
ATR-FTIR spectra analyses. Evaluation of the binding efficiency of each nanoprobe to its corresponding antibody to form the nanoprobes for: (**a**) EpCAM, (**b**) MUC-1 and (**c**) HER-2.

Attenuated total reflection-Fourier transform infrared (ATR-FTIR) spectroscopy was employed in reflectance mode to analyze the IR spectra that correspond to monoclonal antibodies, MNPs, and nanoprobes (MNPs + antibodies) targeting EpCAM, MUC-1 and HER-2 (cell surface membrane proteins). The graph (Fig. 4) displays a differential pattern of spectra, in which the absorption bands are associated with the distinct cell types. The main differences are manifested in the spectral range from 800–1800 cm^−1^, especially the wave numbers of 1703 at 1503 cm^−1^ and 1110 at 900 cm^1^.

**Figure 5:**
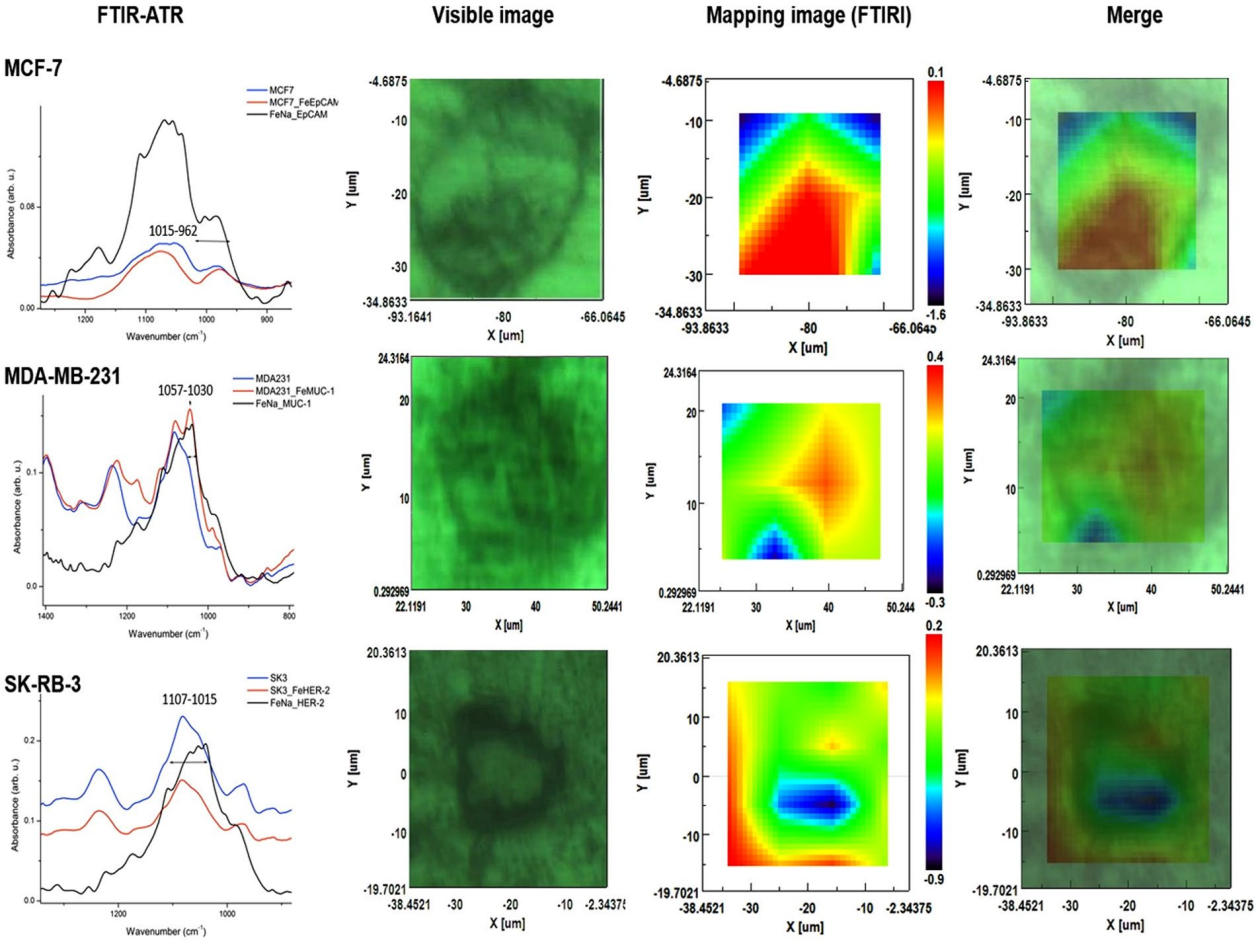
Characterization by ATR-FTIR and FTIRI spectra. The MCF-7, MDA-MB-231 and SK-BR3 breast cancer cell lines were characterized with their corresponding nanoprobe coupled to the respective surface protein (EpCAM, MUC-1 or HER-2) by ATR-FTIR. Blue line = cell line; red line = nanoprobe + cell line; black line = nanoprobe. The location of nanoprobes on the cell membrane was determined by Mapping image (FTIRI) were identified by IR score image as color-coding image red > yellow > blue (greater to smaller concentration of the nanoprobe, in which blue denotes scant or null presence).

The FTIRI of each of the three BC cell lines (MCF-7, MDA-MB-231 and SK-BR-3) shows the map of nanoprobe distribution, where red and blue represent strong and weak absorption of the infrared beam, respectively. The predominantly high absorbance observed on the cell surface membrane correlated with the binding of the nanoprobe to the surface protein. A lower intensity was found inside and around each tumor cell type with the corresponding nanoprobe. EpCAM, MUC-1 and HER-2 nanoprobes, which targeted MCF-7, MDA-MB-231 and SK-BR3 cells, respectively, were detected at 1015–962 cm^−1^, 1057–1030 cm^−1^ and 1107–1015 cm^−1^, respectively (Fig. 5).

### Impedance spectroscopy patterns in breast cancer lines

**Figure 6:**
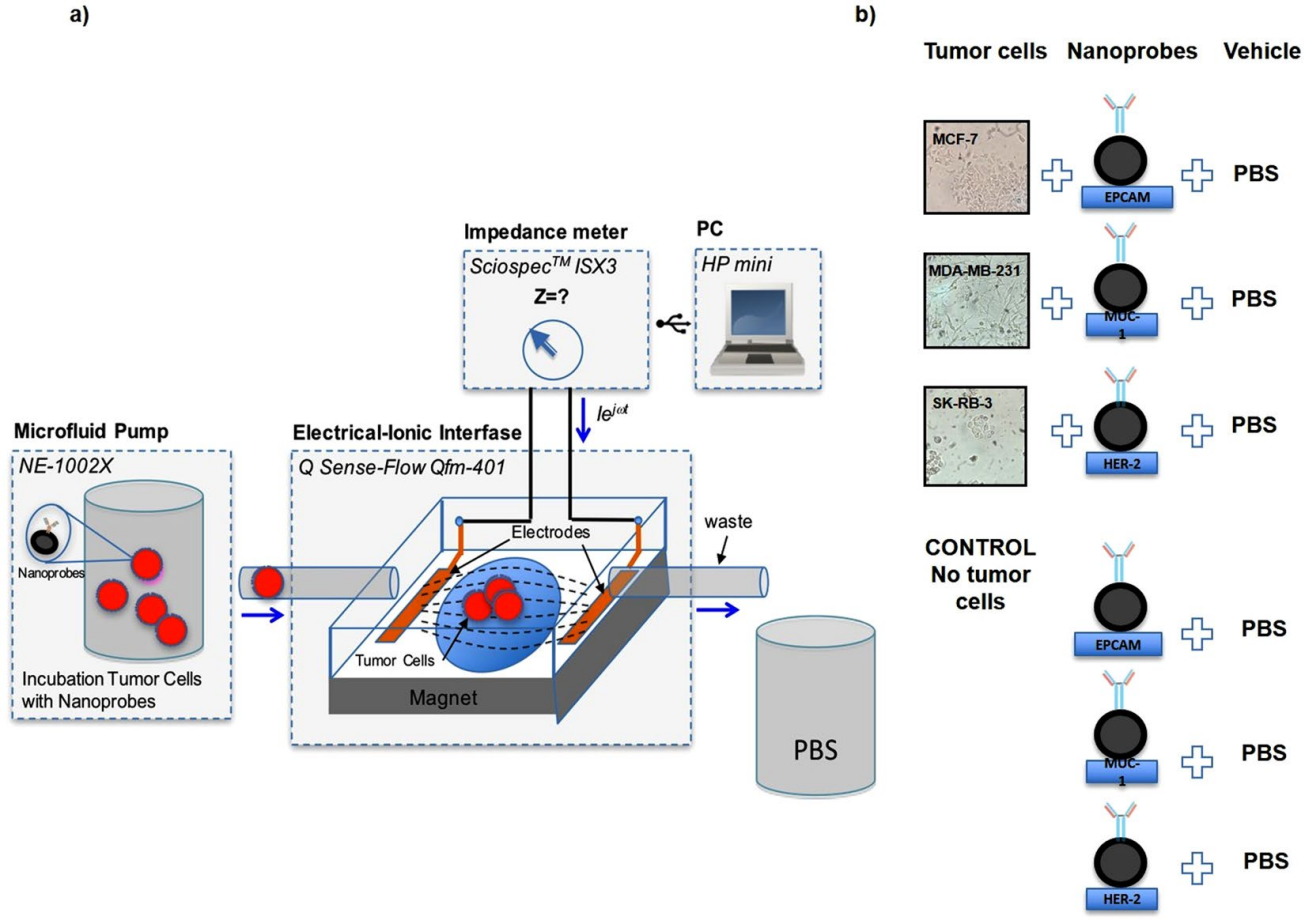
Schematic representation of the biosensor system for tumor cells. (**a**) The system consisted of four modules: an infusion pump, electrical-ionic interface, impedance meter and personal computer. There was a permanent magnet on the bottom of the electrical-ionic module for immunomagnetic insulation and immobilization. (**b**) The two experimental conditions**:** (1) MCF-7, MDA-MB-231 or SK-BR-3 cells were incubated with their respective nanoprobe in 500 μl of PBS; (2) the nanoprobe only (cell-free) was also studied in 500 μl of PBS. Each of three cancer cell lines were assayed in triplicate.

**Figure 7:**
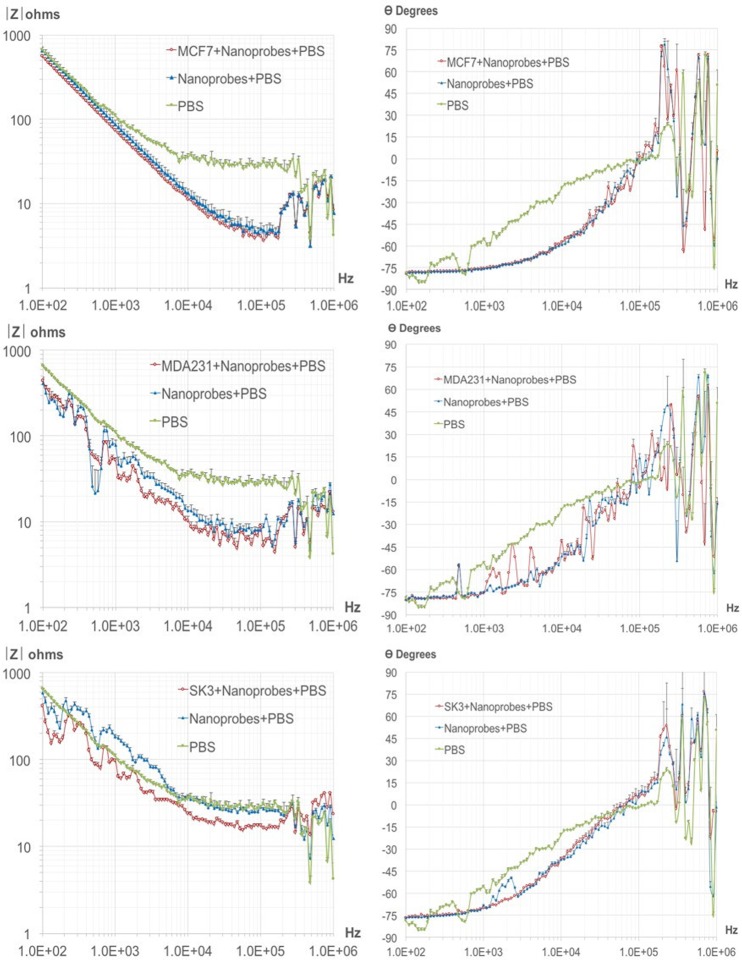
Impedance spectroscopy. Spectra were recorded for the MCF-7, MDA-MB-231 and SK-BR-3 cell lines (at a concentration of 50 cells per 500 μl of PBS) coupled to the corresponding nanoprobe, and for the nanoprobes only.

Impedance spectroscopy was conducted by the methodology depicted in Fig. 6. A graph was constructed to portray the impedance measurements (displaying amplitude and phase) under two conditions: 1) a cell population with the respective nanoprobe in 500 μl of homogenous PBS solution, and 2) the nanoprobe without cells in 500 μl of homogeneous PBS solution (Fig. 7). For each impedance measurement, the 500 μl of PBS contained 50 cells from the corresponding cell line (assays were performed in triplicate).

## Discussion

Currently, the determination of cell surface membrane proteins is part of a series of strategies for detecting circulating tumor cells of epithelial origin. RT-qPCR and flow cytometry were presently carried out to analyze the cell surface membrane of each cell line and identify the existing proteins. RT-qPCR detects messenger RNA. It was followed by flow cytometry, which measures fluorescence intensity (Figs 2 and 3). A distinct expression pattern of proteins was observed for the three BC cell lines, finding predominantly EpCAM in MCF-7, MUC-1 in MDA-MB-231, and HER-2 in SK-BR-3, apparently due to the distinct characteristics of the cells.

Each cell line represents a different pathological stage of BC, with MCF-7 corresponding to the early stage, MDA -MB-231 to the invasive phase, and SK-BR-3 to metastasis. It was found that the more advanced the tumor process, the less the production of characteristic proteins of epithelial origin (e.g., EpCAM) related to cell adhesion receptors. Such proteins maintain cells *in situ* and thus limit their capacity of invasion. The epithelial-mesenchymal transition process gives rise to metastasis, as documented by Walid A. and Tobias M.^25,26^.

The Fourier transform IR (FTIR) spectroscopic technique has been employed to interpret information based on chemically specific IR spectra^27^. It herein served to characterize the proper coupling between nanoparticles and monoclonal antibodies. Differences in absorbance between the nanoparticles and nanoprobe were identified with the ATR-FTIR mode. Nanoparticles displayed characteristic IR stretches of C = O at 1615 cm^−1^ and C-O at 1066 cm^−1^, indicating the presence of D-glucuronic acid. The IR stretch of 90 cm^−1^ obtained from free D-glucuronic acid (1705 cm^−1^) evidenced the chemical bonding of its -COOH group with a nanoparticle, as described by Bony Bradul in relation to lanthanide oxide nanoparticles^28^. According to the A-10 Chemicall protocol for fluidMAG-ARA nanoparticles (methodology section), carbodiimides react with the carboxylate groups from the magnetic beads to afford highly reactive O-acylisourea derivatives, which in turn readily react with amino groups of monoclonal antibodies. Due to the absence of bands in the nanoprobes that correspond to the glucuronic acid-carboxyl group, binding to antibody amino groups had probably occurred. Moreover, the nanoprobe (the antibodies conjugated to nanoparticles) clearly exhibited amide I (1647 cm^−1^) and amide II (1540 cm^−1^) bands. This could be explained by the attachment of antibodies to form the nanoprobe, a hypothesis supported by the absence of the same bands in the FTIR spectrum of Fe_3_O_4_ nanoparticles. These results are in agreement with a report by Yaolin Xu^29^.

ATR-FTIR spectroscopy was carried out to corroborate the suitable coupling between cell lines and their respective nanoprobe. The spectra revealed changes in intensities and frequencies of some absorption bands in spite of the similarities in biochemical profiles. That is, the biochemical profile of a given cell line alone was similar to its profile when bound to the corresponding nanoprobe (MCF-7 coupled to the EpCAM nanoprobe, MDA-231 to the MUC-1 nanoprobe, and SK-BR-3 to the HER-2 nanoprobe). The changes were exhibited at the wave numbers of 962–1015 cm^−1^, 1030–1057 cm^−1^ and 1015–1107 cm^−1^, respectively, suggesting that the nanoprobes were able to bind to the target cell membrane.

Since the images were constructed from fingerprint spectra, it was possible to identify a certain wavenumber on a cell. The ATR-FTIR regions herein described (wavenumber absorption) were used for biochemical mapping with FTIRI, a tool that allows for the construction of images of cell architecture by processing spectral data with a variety of computational algorithms. As expected, the results corroborated the presence of nanoprobes on the cell membrane in each BC cell line. The specific distribution of nanoprobes on the cell surface was established by color-coded scoring of IR images: red > yellow > blue (Fig. 5). These findings correlate with the data from a previous study in which the same imaging tool was used to determine the distribution and chemical structure of biomolecules on tissues and cells^30^.

After completing the biochemical characterization and examining nanoprobe distribution, an impedance spectroscopic analysis was performed on each BC cell line coupled with its corresponding nanoprobe, as well as on the nanoprobe alone. The spectra demonstrate that is possible to identify tumor cells through impedance measurements, evidenced by the capacity of this technique to discriminate between the absence and presence of cells.

The frequency range of 100 Hz–1 MHz was selected to study the electrical properties of the samples in the α and β dielectric dispersion regions, these being the sites for exploring the effect of heterogeneous structure/ionic activity and interfacial polarization of biological membrane systems, respectively^31^. Differences have been found in the amplitude of bioimpedance within the α dispersion bandwidth. At this frequency range, the contribution of cell structure becomes relevant because of the complex structure of the membrane-nanoprobe interface generated by the coupling of tumor cells and MNPs in suspension. Hence, cell structure might play an important role in detection sensitivity.

Impedance magnitude in the absence of cancer cells (PBS + Nanoprobes) for the case of SK-BR-3 cell line rises in the order of PBS measurements, such condition might be explained by the low quanity of nanoprobes required for incubation given the high expression of the predominant surface protein HER-2 with respect to EpCAM and MUC-1 for MCF-7 and MDA-231 cells respectively (see Fig. 3. Determination cell-surface protein expression), thus this particular case represents the lowest concentration of nanoprobes and the magnitude follows the PBS signature, interestingly for the condition with cancer cells present (SK3 + Nanoprobes + PBS) differences respect PBS + Nanoprobes and PBS are much more clear, such finding is also roughly observed for the case of MDA-231 cell line which has an intermediate expression of the surface protein MUC-1 (Fig. 3), finally for the MCF-7 case with the highest nanoprobes quantity required for incubation, magnitude for PBS seems far of nanoprobes conditions, but the sensitivity for cell detection decreases. The observations suggest that as low quantity of nanoprobes are used for detection the impedance sensitivity to detect cells improves, and it might be associated with a percolation effect given MNP´s saturation in the sample.

Variations in phase were not clearly evidenced throughout the frequency range, but a minimal difference was evident in the range of 50–100 KHz. This might represent the capacitive effect of the structural components involved in the interaction of the cell membrane with the nanoprobe, meaning that the coupling of the respective monoclonal antibody and hydrophilic polymers emerges, and the nanoprobe is thus anchored to the electrode surface. The phase becomes positive above 100 KHz, a range associated with an inductive effect in the wiring system. Due to the evident inductive effect, we have omitted discussion of the results obtained in this frequency range.

The whole-system impedance meter and electrical-ionic interface for PBS alone shows dielectric dispersion, mainly at the frequency range below 1 KHz. This likely owes itself to electrode polarization and to the capacitive artifacts integrated into the circuit coupling and wiring. Dielectric dispersion is typical in a two-electrode measurement system, as demonstrated by a previous report^19^. The present data reveal the existence of this phenomenon in the current samples with cellular and nanoprobe content. The main idea here was to evaluate whether a simple two-electrode system provides sufficient sensitivity to enable the differentiation, by using relative impedance measurements, between nanoprobes alone and nanoprobes with BC cells.

**Figure 8:**
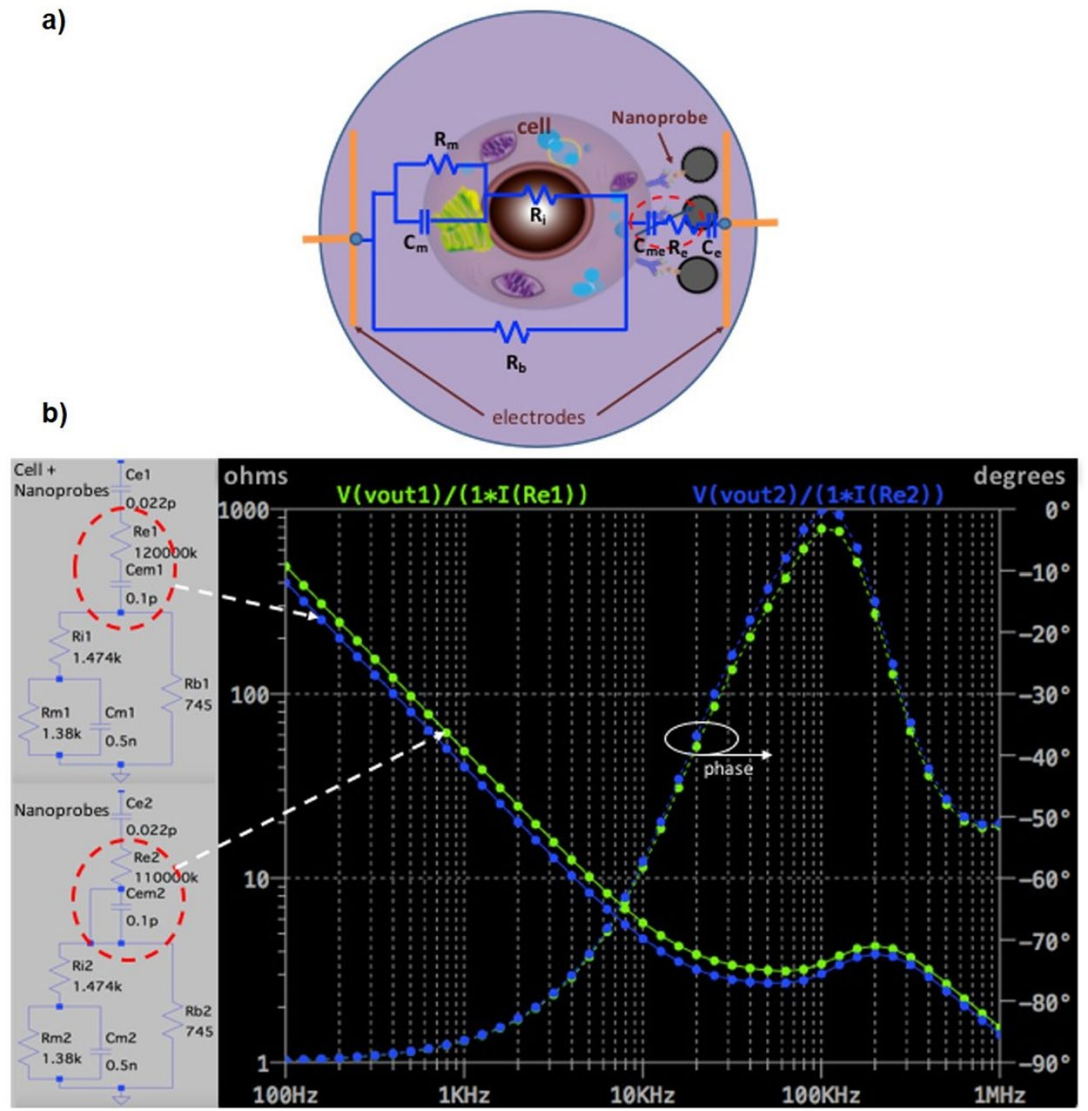
Numerical modelling: (**a**) A simple, typical RC equivalent circuit proposed for a single cell in suspension and coupled to MNPs. (**b**) Frequency response of the RC equivalent circuit calculated by *LTspice IV*.

The present model involved a simple equivalent electrical circuit consisting of a single cell in suspension coupled to MNPs anchored to an electrode surface (Fig. 8a). The equivalent circuit is designed on the basis of a model and experimental values already described^19^. We added resistive and capacitive elements with proposed values to reflect the interactions between the cell membrane, the nanoprobe and the surface electrode. The tumor cell is modeled by R_i_ (1.47 KΩ), R_m_ (1.38 KΩ), C_m_ (0.5 nF) and R_b_ (745Ω) as the intracellular resistance, membrane resistance, membrane capacitance and extracellular resistance, respectively. R_e_ (120 MΩ) and C_me_ (0.1 pF) denote the resistance and capacitance for a composite interaction between the cell membrane and the nanoprobe. Finally, C_e_ (0.022 pF) is the capacitive effect of the nanoprobe on the electrode surface.

A numerical estimation of the frequency response was calculated by *LTspice IV*. In order to make a numerical representation of the effect of the nanoprobe only (without tumor cells), changes were made in resistance (R_e_) and capacitance (C_me_, to reflect the lack thereof). A specific effect was noted, mainly on magnitude at low frequencies (Fig. 8b). The numerical calculations were generally consistent with the experimental findings. Thus, the addition of resistive and capacitive elements to represent the interaction of the cell membrane with the nanoprobe might be key for better understanding the biophysical phenomenon. During this interaction, there is a coupling of the monoclonal antibodies with hydrophilic polymers, as well as a capacitive effect produced by the anchoring of nanoprobes to the electrode surface.

The sensitivity and the signature of the magnitude of impedance spectra are substantially different between the three cancer cell lines, particularly evident in the alfa dispersion bandwidth. The assumption, based on observations by Guofeng Qiao *et al*.^19^, is that the structural heterogeneity between cells lines should emerge. With a standard impedance analyzer driving a four-electrode chamber, three cell suspensions were assessed in the aforementioned study: MCF-10A (normal breast tissue), MCF-7 (early stage BC cells), and MDA-MB-231 (invasive phase BC cells). Using a range of frequencies, variations in electrical properties (e.g., membrane resistance, membrane capacitance and cytoplasm conductivity) were noted when comparing the three cell lines. The authors concluded that these electrical parameters can serve as electrical signatures for the cells and be used for identification (taking into account the physical and biological condition of each cell)^19^. Hence, impedance spectroscopy seems to be a valuable technique not only for detecting cancer cells in the blood, but also for distinguishing between cancer cell types. Accordingly, bioimpedance measurements made with the help of MNPs might be relevant for monitoring of targeted drug delivery, the electroporation process and/or the separation and detection of bacteria.

## Conclusions

The results suggest that impedance spectroscopy was sufficiently sensitive to identify extremely low concentrations of cancer cells (coupled to nanoparticles with magnetic properties) in aqueous solution. This procedure, carried out by using nanoprobes (antibodies coupled to the MNPs) to target proteins on the cell surface membrane of BC cells, represents an economical and practical methodology that will likely prove to be capable of detecting cancer cells in blood.

## Methods

### Breast cancer cell lines

The BC cell lines employed presently (MCF-7, MDA-MB-231 and SK-BR-3) were obtained from the National Institute of Cancerology (Mexico City). They were seeded in a 100 mm culture dish and maintained in Dulbecco’s Modified Eagle Medium F12 (DMEM-F12, Gibco-Bethesda Research Laboratories, Gaithersburg, MD, USA) supplemented with 10% Fetal Bovine Serum and 1% Penicillin–Streptomycin-Amphotericin B (Invitrogen, USA). The cell culture medium was changed every 2–3 days, depending on the cell growth rate. The cells were maintained in an incubator at 37 °C, 5% carbon dioxide (CO_2_) atmosphere and 95% humidity. Each cell line was independently cultured for each of the three biological replicates involved in the experiments. For phase-contrast microscopy, images of live cells growing on the culture dish were collected on an NIKON Eclipse TS100 microscope equipped with a Lumenera Scientific Infinity 1 color digital camera (Fig. 1).

### Differential expression analysis by RT-qPCR

In order to evaluate the gene expression profile of each BC cell line, total RNA was isolated from cultured cells with TRIzol reagent (Life Technologies, Grand Island, NY, USA) by following the manufacturer’s instructions. The quantity and quality of the insolated RNA was determined on a NanoDrop 1000 UV Visible Spectrophotometer (Thermo Scientific). The samples were treated with a DNase RQ1 RNase-free DNase kit (Promega, Cat. #M610A, Madison, WI, USA) to avoid contamination with genomic DNA. The complementary DNA (cDNA) corresponding to each biological replicate was synthesized with an Invitrogen Cloned AMV First-Strand cDNA Synthesis kit (Life Technologies, Cat. #12328-032, USA). Quantitative real-time PCR (RT-qPCR) was performed on the cDNA from each cell line. RT-qPCR primers were designed for EpCAM, Muc-1 and HER-2 as well as for the endogenous gen by employing Applied Biosystems Primer Express software version 3.0 (Table 1).

**Table 1:** Primers used for RTqPCR.

Gene	Sequence 5′-3′
*MUC-1*	S: AAGAACTACGGGCAGCTGGA
	A: TGCCACCATTACCTGCAGAA
*EpCAM*	S: GCTGGCCGTAAACTGCTTTG
	A: TTTTGCTCTTCTCCCAAGTTTTG
*HER-2*	S: ACCTTTCTACGGACGTGGGA
	A: ACCTCTCGCAAGTGCTCCAT
*β-Actin*	S: CGGGAGATTGTGCGAGATGT
	A: GGAAGCGTTCATTCCCAATG

Gene amplification was carried out on ABI PRISM 7000 Sequence Detection System-SDS device, version 1.1, with the SYBER Green PCR Master Mix kit (Applied Biosystems) as the method of monitoring the amplification of the product during each reaction cycle. *β-Actin* served as the endogenous gene for the examination of *HER-2*, *EpCAM* and *MUC-1*. The RT-qPCR reaction was verified and validated by the Rasmussen equation^32^. Relative expression was calculated by the ΔΔCT method with the arithmetic formula 2^−ΔΔCT^^33^.

### Immunophenotyping of breast cancer cell lines by flow cytometry

Protein expression was examined by flow cytometry assays to confirm the molecular profile of surface markers for each BC cell line (MCF-7, MDA-MB-231 and SK-BR-3). The distribution of cell surface proteins (EpCAM, MUC-1 and HER-2, respectively) could be determined by the use of monoclonal antibodies.

MCF-7, MDA-MB-231 and SK-BR-3 cells were removed from the culture dish, washed, suspended at 10^6^ cells per 1 ml tube and incubated for 30 minutes in the dark at room temperature. During incubation, they were labeled with the following monoclonal antibodies: phycoerythrin (PE) anti-human CD227 for MUC-1 (Biolegend, Cat. #355603, San Diego, CA, USA), allophycocyanin (APC) anti-human CD326 for EpCAM (Biolegend, Cat. #324207) and brilliant violet 421 anti-human CD340 for HER-2 (Biolegend, Cat. #324420). Subsequently, the excess of non-coupled antibodies was removed with phosphate buffer (PBS 1 X) and unspecified joints were blocked with 1% albumin in PBS (PBA). Finally, the cells were fixed with 1% formaldehyde. From each BC cell line, 10,000 events were acquired in a FACS Aria III (Becton Dickinson) flow cytometer and analyzed on Flow Jo (v10.1) software.

### Immuno-magnetic coupling

Three nanoprobes were created by coupling fluidMAG-ARA nanoparticles GmbH (Chemicell, Cat. #4115-5, Berlin, Germany) to each of the following monoclonal antibodies: anti-human CD36 (EpCAM, Biolegend, Cat. #324202), anti-human CD227 (MUC-1, Biolegend, Cat. #355602) and anti-human CD340 (erbB2/HER-2, Biolegend, Cat. #324402, Biolegend). Each nanoprobe targeted its corresponding cell surface membrane protein. These nanoparticles were magnetic iron oxides (in aqueous dispersion) consisting of a core of magnetite (Fe_3_O_4_) covered with hydrophilic polymers. The latter covering protects the particles against aggregation by foreign ions. The nanoparticles also contain glucuronic acid-carboxyl as a functional group, which allows for the covalent immobilization of biomolecules useful for binding to nanoparticles.

The conjugate was formed with carbodiimide crosslinker chemistry, thus producing covalent coupling as described in the A-10 Chemicell protocol for fluidMAG-ARA nanoparticles. Briefly, 10 mg of fluidMAG-ARA nanoparticles were washed twice with 1 ml of 0.1 M 2-(N-Morpholino) ethanesulfonic acid (MES) as a buffer, in the presence of a magnetic separator. Nanoparticles were then placed in a tube containing 10 mg of 1-ethyl-3-[3-(dimethylamino)propyl]carbodiimide dissolved in 0.25 ml MES and incubated for 10 min at room temperature. Upon completion of this period, activated nanoparticles were washed twice and suspended in 1 and 0.25 ml MES. Fifty micrograms of each of the three monoclonal antibodies were incubated in a tube containing activated nanoparticles being gently mixed for 2 h at room temperature. Finally, the nanoparticles were washed 3 times with 1 ml of phosphate-buffered saline (PBS) and resuspended in 200 μl of blocking/storage buffer. The final nanoparticle–antibody ratio in the nanoprobes was 10 mg per 50 μg.

### ATR-FTIR of the nanoprobe alone and the nanoprobe plus each BC cell line

The coupling between nanoparticles and the corresponding monoclonal antibodies was assessed by the carbodiimide method (A-10 Chemicell Protocol). Accordingly, the ATR-FTIR spectroscopic analysis was performed on each of three conditions, placing 2 μl of the following sample on a diamond crystal: (1) the antibody (for EpCAM, MUC-1 or HER-2), (2) the nanoparticles, and (3) the nanoprobes (nanoparticle + antibody). A FTIR spectrometer (Jasco 6600) was employed to make the measurements in Attenuated Total Reflection mode (ATR) with a diamond crystal. The instrument was set for a fixed spectral resolution of 4 cm^−1^, a spectral range from 400–4000 cm^−1^ and 120 scans.

ATR-FTIR spectra were also obtained to corroborate the nanoprobe recognition of a specific cell line. Briefly, the MCF-7, MDA-MB231 and SK-BR3 cell lines were cultured and incubated until reaching 80% confluence. Subsequently, the cells were washed twice with isotonic saline solution and removed mechanically. After being centrifuged and resuspended in 50 μl of isotonic solution, 2 μl of the cells (from each cell line) were used as the control (in the absence of the nanoprobe), and 25 μl were incubated for 15 minutes with the corresponding nanoprobe. Finally, analysis of 2 μl of the cell sample was carried out by FTIR-ATR in order to verify the suitable coupling between the nanoprobe and the cells, as aforementioned.

### Biochemical mapping

Antibody-conjugated nanoparticles were evaluated with biochemical mapping (FTIRI) to determine their targeting efficiency and specificity for EpCAM, HER-2 or MUC-1 in positive cell lines. Each BC cell line (MCF-7, MDA-MB231 and SK-BR3) was independently cultured and placed on the surface of a gold-coated slide, followed by incubation under previously described conditions until reaching 80% confluence. Subsequently cells were washed twice with isotonic saline solution to remove the medium. The slides were then divided into two parts, with one side used as the control (without nanoprobes) and the other incubated with the specific nanoprobe for 15 minutes. Finally, the slides were washed and dried at room temperature for about 10 minutes to remove excess water.

FTIRI was conducted on a FTIR microscope (Jasco IRT-5200) using a 32X Cassegrain objective. The equipment was fitted with a liquid nitrogen-cooled MCT (Mercury, Cadmium, Tellurium) detector coupled to a FTIR spectrometer (Jasco 6600). The absorbance spectrum was acquired in reflectance mode at a spectral resolution of 4 cm^−1^ with 60 scans in the spectral range of 400–4000 cm^−1^. Biochemical images were obtained by automated mapping of multiple points (IQ mapping) on the FTIR microscope. The bands examined with this method corresponded to the three nanoprobes. The data for each spectral band was portrayed in two-dimensional images, representing the density distribution of each nanoprobe on the cell membrane of the respective BC cell line.

### Impedance spectroscopy measurements

The biosensor system for tumor cells consisted of an infusion pump, an electrical-ionic interface, an impedance meter and a personal computer. The infusion pump (NE-1002X) was adapted with a 1 ml syringe to control the infusion of the analytes towards the electrical-ionic interface through a capillary tube. The electrical-ionic interface was produced in a microfluidic chamber (*Q Sense-Flow Qfm-401, Biolin Scientific*^*TM*^) that housed a 14 mm diameter quartz crystal (QSX3.1, *Biolin Scientific*^*TM*^) with a 9 mm diameter gold surface. In this module, the tumor cell anchoring process was generated by magnetism. Additionally, the interconnection of the gold surface and the chamber in a two-electrode configuration enabled the bioimpedance measurements. The impedance meter (ScioSpec, ISX-3, Germany) allowed for interaction with the electrical-ion interface by injecting a 100 mV peak potential difference and then measuring the current to estimate the impedance of the system. The PC (HP mini 110-1150LA PC, HP Inc.) was used to program ScioSpec and store the data in an appropriate format (Fig. 6a).

In order to identify the impedance resulting from the presence of tumor cells, bioimpedance spectroscopy measurements were made for MCF-7, MDA-MB-231 and SK-BR-3 cells incubated with their respective nanoprobe in 500 μl of PBS. For each cell line, the aforementioned measurement was compared to that made for the corresponding nanoprobe only (in 500 μl of PBS, without cells).

The nanoprobe employed for each cell line targeted the predominant cell surface protein, which was previously confirmed by RT-qPCR and flow cytometry analyses. The quantity of the nanoprobe used in each assay was selected by considering the concentration of antibody per magnetic nanoparticle (50 μg/10 mg) according to the protocol of fluidMAG-ARA, and the amount of antibody per million cells recommended by the product data sheet of each antibody (Table 2).

**Table 2:** Amount of antibody recommended per million cells.

Antibody	Amount per million cells
Anti-human CD326 (EpCAM)	≤0.5 μg per 10^6^ cells.
Anti-human CD227 (MUC-1)	≤1.0 μg per 10^6^ cells.
Anti-human CD340 (erbB2/HER-2)	≤0.125 μg per 10^6^ cells.

Subsequently, the solution was diluted to a concentration of approximately 50 cells in 500 μl PBS. The experiments were conducted in triplicate for each BC cell line (Fig. 6b). In addition, PBS (the carrier of the samples) was evaluated as the reference condition for the electrode-ion interface with impedance/parasitic capacitance effects. Each condition was independently infused through the tube for 15 minutes, thus exposing it to a magnetic field. Impedance spectra were recorded, obtaining the signal between each of the intervals of 1024 average values per second to determine magnitude and phase. Following the measurement of each condition, the system was cleaned with 500 μl PBS (Supplementary Data).

### Statistics

The data, expressed as the mean ± SEM, were analyzed on GraphPad Prism software version 5.01. The Kruskal-Wallis test was applied to compare the gene expression between BC cell lines (considering the results of three independent biological replicates in each case). Significant differences were established by Dunn´s post hoc test. A p-value < *0.05* was considered statistically significant.

## Supplementary information

Dataset 1
